# Contribution of Membrane Lipids to Postsynaptic Protein Organization

**DOI:** 10.3389/fnsyn.2021.790773

**Published:** 2021-11-23

**Authors:** Manon Westra, Yolanda Gutierrez, Harold D. MacGillavry

**Affiliations:** Cell Biology, Neurobiology and Biophysics, Department of Biology, Faculty of Science, Utrecht University, Utrecht, Netherlands

**Keywords:** synapse, membrane, lipid, membrane organization, synaptic plasiticity, synaptic plasma membrane

## Abstract

The precise subsynaptic organization of proteins at the postsynaptic membrane controls synaptic transmission. In particular, postsynaptic receptor complexes are concentrated in distinct membrane nanodomains to optimize synaptic signaling. However, despite the clear functional relevance of subsynaptic receptor organization to synaptic transmission and plasticity, the mechanisms that underlie the nanoscale organization of the postsynaptic membrane remain elusive. Over the last decades, the field has predominantly focused on the role of protein-protein interactions in receptor trafficking and positioning in the synaptic membrane. In contrast, the contribution of lipids, the principal constituents of the membrane, to receptor positioning at the synapse remains poorly understood. Nevertheless, there is compelling evidence that the synaptic membrane is enriched in specific lipid species and that deregulation of lipid homeostasis in neurons severely affects synaptic functioning. In this review we focus on how lipids are organized at the synaptic membrane, with special emphasis on how current models of membrane organization could contribute to protein distribution at the synapse and synaptic transmission. Finally, we will present an outlook on how novel technical developments could be applied to study the dynamic interplay between lipids and proteins at the postsynaptic membrane.

## Introduction

Experience-dependent modulation of synaptic connections in the brain underlies complex cognitive processes such as learning and memory. In particular, activity-dependent changes in the postsynaptic organization are thought to be essential for the expression of the long-term changes in the efficiency of synaptic transmission that underlie memory formation (Martin et al., 2000; Takeuchi et al., 2014). Indeed, recent super-resolution microscopy studies demonstrated that the positioning of synaptic scaffolding molecules and receptors anchored at the postsynaptic density (PSD) is tightly controlled at the nanoscale and is adjusted by synaptic activity (Fukata et al., 2013; MacGillavry et al., 2013; Nair et al., 2013; Tang et al., 2016; Goncalves et al., 2020). Specifically, subsynaptic clusters of receptors, or nanodomains, in the synaptic membrane enriched in AMPA- or NMDA-type glutamate receptors (AMPARs and NMDARs) and scaffolding molecules were found to be aligned with the presynaptic glutamate release site to optimize synaptic transmission (Tang et al., 2016; Li et al., 2021). However, how these nanodomains are formed and modulated during synaptic plasticity remains unknown.

Despite synaptic receptors being integral membrane proteins that are embedded in the lipid bilayer, the contribution of lipids to synaptic organization and functioning remains poorly understood. Nevertheless, lipids are the most abundant components of the brain and lipid dysregulation is thought to underlie several cognitive disorders (Kanungo et al., 2013; Martín et al., 2014; Pérez-Cañamás et al., 2017; van der Kant et al., 2019). Interestingly, synapses are enriched in specific lipid species such as cholesterol and sphingolipids (Breckenridge et al., 1972) and other less abundant components, such as phosphoinositides. This unique lipid composition can have various important consequences for synapse organization and functioning. For instance, lipids can control compartmentalization and proper positioning or activation of critical synaptic protein complexes (Haucke and Di Paolo, 2007; Arendt et al., 2010; Dotti et al., 2014; Brachet et al., 2015). Moreover, changes in lipid composition determine membrane viscosity, thereby directly controlling the mobility and lateral diffusion of membrane molecules. Indeed, the particular composition of the lipid bilayer strongly favors the maintenance of a heterogeneous spatial organization of membrane lipids and associated proteins (Ingólfsson et al., 2017; Fitzner et al., 2020). The unique composition and structure of the synaptic membrane is therefore predicted to directly impact the activity-dependent changes in protein organization at synapses, ultimately controlling synaptic physiology and brain function.

In this review we will focus on the contribution of the postsynaptic plasma membrane to synapse organization and neuronal function. We will discuss our current understanding of the lipid composition of the synaptic membrane, consider intrinsic and extrinsic factors that influence membrane organization and lastly, we will highlight technical advances that can be used to further study the role of the membrane in postsynaptic organization.

## The Neuronal and Synaptic Lipidome

The composition of the plasma membrane is significantly different between cell types, is adjusted during developmental stages and can adapt in response to environmental changes. We are only beginning to understand how this dynamic diversity in lipid composition influences cellular functions but it is becoming clear that the heterogeneity in lipid composition directly determines physical properties of the membrane and is important for key cellular processes.

**FIGURE 1:**
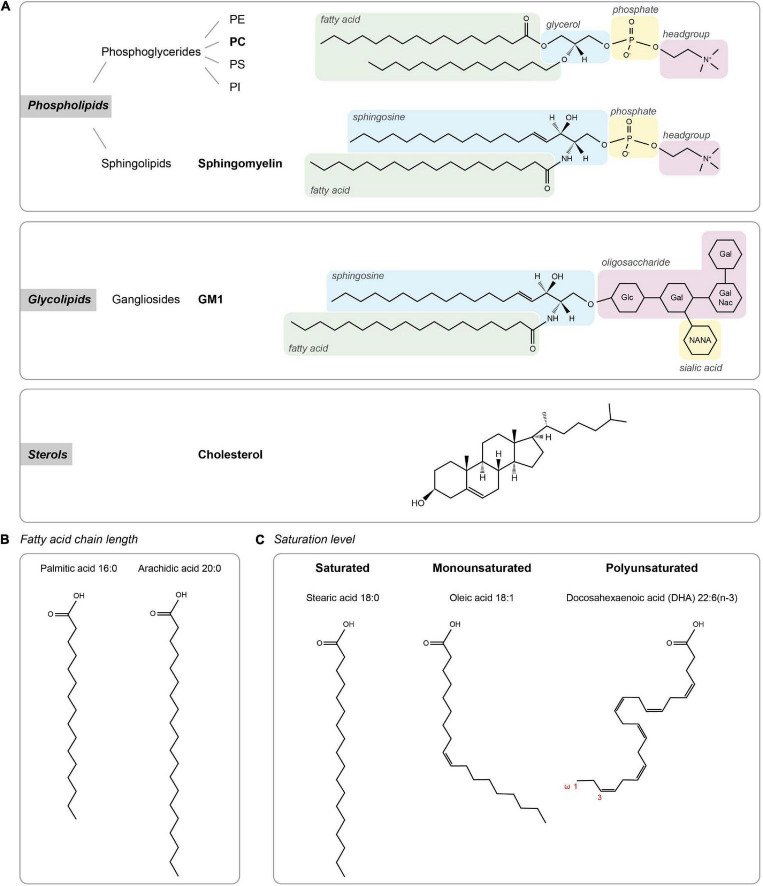
Lipid types. **(A)** The three major classes of membrane lipids (phospholipids, glycolipids, and sterols) with an example lipid structure (bold) for each. Glc – D-glucose, Gal – D-galactose, NANA – N-actylneuraminic acid, and GalNac – *N*-acetyl-D-galactoseamine. **(B,C)** Acyl chain composition. **(B)** Fatty acid chain length for palmitic acid and arachidic acid. **(C)** Lipid structures of lipids with different saturation levels (stearic acid, oleic acid, and docosahexaenoic acid).

Cellular membrane lipids are amphipathic molecules with a characteristic polar headgroup and long hydrophobic fatty acid tails causing them to spontaneously form a thin lipid bilayer (Figure 1). Lipids can be categorized based on their head groups, fatty acid chain lengths and degree of saturation. The three major classes of membrane lipids are phospholipids, glycolipids, and sterol (Figure 1A). Phospholipids form the vast majority of lipids in plasma membranes (>50%), with a small contribution of glycolipids (<5%). Cholesterol constitutes 25–35% of the membrane lipids and provides rigidity to the plasma membrane. Together, the phospholipids phosphatidylethanolamine (PE), phosphatidylcholine (PC), phosphatidylserine (PS), and sphingomyelin constitute more than half the mass of lipids in most mammalian membranes.

Advances in lipidomic profiling have enabled the precise identification and quantification of lipid species in tissues. These approaches revealed that lipid composition of the brain is highly distinct from other tissues with relatively high levels of cholesterol and polyunsaturated fatty acids (PUFAs; Bozek et al., 2015; Fitzner et al., 2020). Interestingly, comparison between species revealed that this diversity rapidly expanded in primates, linking brain lipidome complexity to the evolution of higher cognitive brain functions (Bozek et al., 2015). Further analysis of cell-type specific lipid profiles revealed that neurons are particularly enriched in cholesterol and ceramide (Fitzner et al., 2020).

Several studies have investigated the lipid composition of synaptic plasma membranes isolated using zonal centrifugation from adult rat brain (Cotman et al., 1969; Breckenridge et al., 1972; Igbavboa et al., 2002; Tulodziecka et al., 2016). The major lipid types in synaptic membranes are cholesterol, phospholipids and gangliosides, with PE and PC as the most abundant phospholipids (Cotman et al., 1969; Igbavboa et al., 2002). Compared to the whole brain, the fraction of glycolipids in synaptic membranes seems lower while sphingomyelin seems more abundant (Cotman et al., 1969). Interestingly, although sphingomyelin is detected at low levels, in contrast to other membranes, in synapses sphingomyelin is composed of almost exclusively stearic (18:0) acid (Breckenridge et al., 1972). When looking more closely at the fatty acid composition, it was found that the synaptic plasma membrane is particularly enriched in PUFAs (Cotman et al., 1969; Breckenridge et al., 1972; Igbavboa et al., 2002). Particularly high levels of docosahexaenoic acid (DHA) in PE and PS phospholipids were detected, which is a striking difference compared to the plasma membrane composition of other tissues. A recent comprehensive lipidomic study showed that the lipid composition of the PSD membrane evolves with development (Tulodziecka et al., 2016), with key species such as cholesterol progressively increasing during development. Additionally, glycosphingolipid levels are developmentally regulated and increase throughout postnatal life (Ngamukote et al., 2007).

It is worth noting, however, that several technical limitations prevent forming a comprehensive characterization of the absolute synaptic plasma membrane lipidome with existing biochemical approaches. Whereas synaptosomal preparations contain a mixture of presynaptic membranes, like synaptic vesicles, and other organellar membranes, isolation of PSD plasma membrane relies on the use of non-ionic detergents that can influence the extracted lipid content. Nevertheless, despite differences in absolute numbers of certain lipid species, namely cholesterol and glycosphingolipids, relative compositional changes in response to specific conditions can reliably be detected (Tulodziecka et al., 2016).

## Membrane Composition Dictates Its Organization: Intrinsic Factors

The distinct lipidomic profile of neuronal membranes is likely to influence key neuronal functions. Particularly at synapses, the specific lipid composition could contribute to the heterogeneous nanodomain organization of receptors. However, testing the precise contribution of individual lipids to membrane organization and function in neurons remains technically challenging. Nevertheless, pioneering studies in model membrane systems have characterized the unique biophysical properties of individual lipid species and revealed that these intrinsic properties determine important organizational properties of membranes. We will first provide a brief overview of the general concepts and models of membrane organization and then discuss how these could be incorporated in our current understanding of synapse organization. For more extensive reviews on membrane organization, we refer to a few excellent recent reviews (Sezgin et al., 2017; Jacobson et al., 2019; van Deventer et al., 2021).

### Contribution of Biophysical Properties of Lipids to Membrane Organization

The classic fluid mosaic model (Singer and Nicolson, 1972) was the first to conceptualize and explain experimental observations on the fluidic nature of the plasma membrane. It emphasized that the two-dimensional lipid bilayer is liquid, and that membrane fluidity is the key driver that allows the heterogeneous mixing of lipids and membrane proteins. Almost 50 years later, this model is still valid and has clear relevance for our current thinking on membrane organization. The fluidity of membranes is a key determinant of the diffusion rate of lipids and transmembrane proteins in the membrane as formalized in the hydrodynamic model proposed by Saffman and Delbrück (1975). Membrane fluidity is largely determined by acyl chain composition of membrane lipids (Figures 1B,C). First, longer acyl chains have a larger surface area available for Van der Waals interactions with neighboring acyl chains, reducing membrane fluidity. Second, while straight saturated acyl chains can be efficiently packed closely together, the kink in the hydrocarbon chain of unsaturated acyl chains prevents efficient packing and thus helps to maintain membrane fluidity. Another important determinant of fluidity is cholesterol, which generally promotes packing of lipids.

**FIGURE 2:**
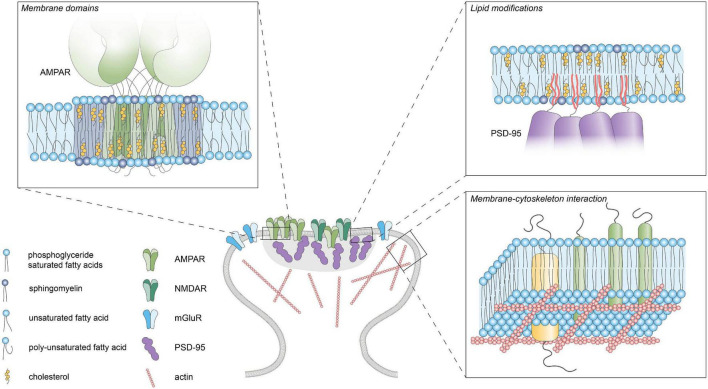
Lipid-protein interactions. Schematic diagram of the different lipid-protein interactions within the postsynaptic plasma membrane. Membrane domains enriched in cholesterol, glycosphingolipids and saturated lipids can facilitate clustering of membrane proteins by influencing fluidity and thickness of the membrane. Reversible palmitoylation can translocate proteins to the plasma membrane. Actin-linked transmembrane proteins can act as “pickets” and hinder the diffusion of lipids and proteins within the membrane.

Driven by their intrinsic biophysical properties, long saturated acyl chains and cholesterol tend to segregate into tightly packed, liquid-ordered (Lo) phases, whereas unsaturated acyl chains preferentially accumulate in liquid-disordered (Ld) phases. This phase behavior has been studied extensively in synthetic model membranes and has also been observed in giant plasma membrane vesicles (GPMVs) derived from living cells (Baumgart et al., 2007). These observations have greatly influenced the lipid raft theory proposing the existence of ordered lipid domains enriched in cholesterol and glycosphingolipids that facilitate the clustering of specific membrane proteins and associated signaling complexes to form dynamic signaling platforms (Simons and Ikonen, 1997; Figure 2). This theory has been investigated vigorously by biochemical methods that extract detergent-resistant membranes (DRMs) enriched in glycosphingolipids and cholesterol (Brown and Rose, 1992). However, because of technical caveats associated with these biochemical approaches and the absence of direct visualization of lipid rafts in living cells, this theory has gained considerable criticism (Pike, 2009; Levental et al., 2020). Nevertheless, in general, rafts are considered to constitute rather small (20–200 nm) and transient membrane domains (Pike, 2006; Eggeling et al., 2009) and considerable attention has been devoted to characterize raft-promoting lipids such as cholesterol and glycosphingolipids in different cellular systems. In contrast, the role of (poly-) unsaturated fatty acids in membrane organization is less well understood. However, these lipids are increasingly recognized as drivers of membrane domain formation (Wassall and Stillwell, 2009). Particularly, studies using GPMVs indicate that polyunsaturated lipids (especially DHA) promote the formation and stabilization of ordered membrane domains by increasing the phase difference in ordering (Levental et al., 2016, 2017).

Variations in membrane thickness can also have profound consequences for the organization of transmembrane proteins. The thickness of the membrane is primarily determined by the acyl chain properties of the lipids, with longer, saturated chains forming thicker membranes. When the hydrophobic transmembrane segment of a protein does not match the hydrophobic thickness of the membrane, a so-called hydrophobic mismatch will occur. To compensate for such hydrophobic mismatches, lipids with matching chain lengths will preferentially surround the transmembrane segment causing local variations in the lateral distribution of lipids. Also, the protein can adapt its orientation or conformation to match the thickness (de Planque et al., 2001), or even undergo aggregation to minimize the mismatch. Hydrophobic matching has therefore been proposed as a mechanism that drives self-assembly of domains consisting of transmembrane proteins and lipids with similar hydrophobic length (Mouritsen and Bloom, 1984; Anderson and Jacobson, 2002). Indeed, computational simulations and experimental analysis in model membranes and cells indicate that hydrophobic matching could promote the lateral segregation of proteins and lipids which is further modulated by cholesterol (Kaiser et al., 2011; Diaz-Rohrer et al., 2014). The mattress model by Mouritsen and Bloom (1984) proposes that hydrophobic mismatch promotes lateral segregation in the membrane such that lipids and proteins self-organize in domains of similar hydrophobic thickness. This has for instance been found to underlie the segregation into functionally distinct membrane domains of two related SNARE proteins, Syntaxin-1 and -4 (Milovanovic et al., 2015). Whether such mechanisms underlie the compartmentalization of postsynaptic transmembrane proteins has not been studied yet.

The concepts and models discussed here conceptualized many of the observations on membrane organization in synthetic and cellular membrane models. However, it is becoming increasingly clear that these models are not universal and the factors determining membrane heterogeneity are highly interdependent (Bernardino de la Serna et al., 2016). Both fluidity and membrane thickness can lead to lateral heterogeneity in the membrane. Particularly, in cellular membranes interactions between lipids and membrane proteins seem dominant in determining membrane domain formation. Thus, the nature of membrane domains, i.e., their spatial dimensions and lifetimes are likely to be highly dependent on the specific subcellular composition of the membrane. Indeed, the current goal of the field is to understand how the interplay between the biophysical properties of lipids and membrane proteins orchestrates membrane organization.

## Intrinsic Determinants of Postsynaptic Membrane Organization

At excitatory synapses, the density of glutamate receptors is a direct determinant of synaptic strength. Mechanisms that control the retention and positioning of receptors have therefore gained tremendous interest. Scaffold proteins in the PSD form a structural platform that anchor receptors via intricate networks of protein-protein interactions. Nevertheless, concepts in membrane biology pose a central role for the intrinsic capacity of lipids to self-organize and form functional membrane domains. In the following sections we discuss how the unique composition of the postsynaptic membrane suggests that synapses actively maintain and perhaps adjust this composition to instruct the organization and function of synaptic protein components.

### Fluidity Controls Receptor Diffusion

Lipidomics studies consistently point out that the brain and particularly synaptic membranes are enriched in both cholesterol and PUFAs. How does this specific composition, with high concentrations of lipids that have opposing effects on lipid ordering and membrane fluidity, influence fluidity at the postsynaptic membrane? Commonly, quantification of the mobility of transmembrane proteins using fluorescence recovery after photobleaching or single-molecule tracking approaches is taken as an estimate of membrane fluidity. However, synaptic membrane proteins are mostly either directly anchored to scaffold proteins or are slowed down in their diffusion due to the relative high density of proteins at the synapse (Li and Blanpied, 2016; Li et al., 2016). In fact, the diffusion of even small transmembrane proteins that are unable to bind synaptic scaffolds is severely influenced by local, subsynaptic variations in cytoplasmic protein density (Li and Blanpied, 2016). Measures of protein mobility do therefore not directly report on the fluidity or ordering of lipids within the membrane itself but are the result of a complex interplay of many different factors. Nevertheless, single-molecule tracking studies showed that even the diffusion rates of fluorescently labeled lipids that are not specifically enriched at synaptic sites are significantly reduced in the postsynaptic membranes compared to extrasynaptic regions (Renner M. L. et al., 2009). However, more direct measures of membrane fluidity using for instance environment-sensitive dyes would be of interest. As an alternative, recently developed computational approaches now allow investigation of the dynamic interplay between lipids and membrane proteins at high spatiotemporal resolution (Ingólfsson et al., 2016). In particular, coarse-grained molecular dynamics simulations allow accurate predictions of how mixtures of lipid species are organized. In a recently developed model of a “brain-like plasma membrane” it was found that while the high concentration of cholesterol leads to an overall increase in acyl chain ordering, the fluidizing effect of high levels of tail unsaturation appears to balance this out (Ingólfsson et al., 2017). Interestingly, however, the extent of ordering in the case of brain membranes was unequally divided over the inner and outer membrane leaflets, with the brain membrane showing distinctively more ordering in the outer leaflet. Also, diffusion rates of lipids were on average 40% lower in brain membranes. Comparable to earlier models of cellular membranes, considerable heterogeneity in the lateral distribution of lipids was found, with more but smaller and more transient cholesterol domains in the brain membrane. These simulations thus provide an unprecedented high-resolution snapshot of how the plasma membrane of neurons could be organized, and it will be of interest to expand these models to test how the high molecular density of integral and membrane-associated proteins at the synapse will influence and interact with this specific composition of lipids.

### Synapses Have Raft Properties

The enrichment of cholesterol and sphingolipids at synaptic membranes and the computational simulations suggest that the postsynaptic membrane could have confined regions reminiscent of lipid rafts. Indeed, DRMs isolated from whole brain contain key components of excitatory synapses, most notably PSD-95, as well as glutamate receptors and interacting proteins (Perez and Bredt, 1998; Brückner et al., 1999; Suzuki et al., 2001, 2011; Hering et al., 2003; Ma et al., 2003; Besshoh et al., 2005; Hou et al., 2008; Delint-Ramirez et al., 2010). Moreover, rafts can be isolated from synaptic membrane fractions (Suzuki et al., 2001, 2011; Besshoh et al., 2005) and ChTx (cholera toxin) labeling overlaps with PSD-95 staining, indicating the presence of raft-like structures at the PSD (Perez and Bredt, 1998; Brückner et al., 1999; Suzuki et al., 2001; Hering et al., 2003; Hou et al., 2008). At the ultrastructural level, electron cryotomography showed that GM1-positive raft-like membranes were frequently found associated, preferentially with adult PSDs (Suzuki et al., 2001; Besshoh et al., 2005; Swulius et al., 2012), consistent with the developmental increase in raft-promoting lipids at synaptic membranes (Tulodziecka et al., 2016). Further, immuno-EM studies demonstrated the presence of raft markers such as flotilins at the PSD (Suzuki, 2002; Hering et al., 2003), that were also shown to interact with NMDAR subunits (Swanwick et al., 2009). All these data thus suggest that raft-like domains exist within the postsynaptic membrane, perhaps compartmentalizing specific receptor complexes (Perez and Bredt, 1998; Brückner et al., 1999; Suzuki et al., 2001; Hering et al., 2003; Allen et al., 2007; Hou et al., 2008). Indeed, PSD-95-NMDAR complexes isolated from raft fractions were enriched in a different complement of signaling molecules than those isolated from PSD or soluble fractions (Delint-Ramirez et al., 2010). The association of NMDARs with raft vs. non-raft domains has been shown to be regulated for instance during spatial memory formation (Delint-Ramírez et al., 2008) and ischemia (Besshoh et al., 2005) indicating that the association of synaptic receptors with specific membrane domains can be dynamic and regulated by synaptic activity.

Thus, although lipid raft characterization relies on biochemical procedures that may occlude investigation of more complex membrane dynamics, evidence gathered through these and other experimental means clearly points toward the existence of a heterogeneous distribution of different components in the postsynaptic membrane. However, it remains difficult to assess how individual lipid types contribute to this heterogeneity. In large part this is difficult because the behavior of individual lipids is highly dependent on the environment. For example, while PUFAs might form disordered membrane domains, they could also contribute to stabilize ordered membrane domains (Wassall and Stillwell, 2009; Levental et al., 2016). Additionally, the interactions of lipids with proteins provide an extra layer of complexity that could underlie the lateral distribution of postsynaptic membrane components.

### Lipids Modulate Synaptic Transmission

Consistent with the notion that lipid rafts are important for regulating NMDAR function, interfering with membrane cholesterol levels was shown to perturb NMDAR-dependent calcium responses as well as LTP (Koudinov and Koudinova, 2001; Frank et al., 2004, 2008; Kotti et al., 2006; Maggo and Ashton, 2014; Guo et al., 2021). More specifically, cholesterol depletion was reported to decrease the open probability of NMDARs and reduce the fraction of synaptic immobile NMDARs (Korinek et al., 2015, 2020). Furthermore, cholesterol reduction increased basal internalization of AMPARs (Hering et al., 2003) and the mobility of slow diffusing molecules within the synapse (Renner M. et al., 2009). In addition, treatment with statins (inhibitors of cholesterol synthesis) impaired recognition and working memory (Maggo and Ashton, 2014; Guo et al., 2021). Cholesterol replenishment could rescue impaired LTD resulting from cholesterol loss in aged mice, also improving hippocampal learning and memory (Ledesma et al., 2012; Martin et al., 2014). On the contrary, other studies reported enhancement of LTP and hippocampal-dependent learning and memory after cholesterol reduction, while adding cholesterol impaired LTP (Li et al., 2006; Mans et al., 2010; Brachet et al., 2015). These conflicting results could be explained by a dose-dependent effect of cholesterol (Baytan et al., 2008; Wang and Zheng, 2015). To untangle these effects, several studies have looked at it from a different perspective: what influence does glutamatergic synaptic transmission have on cholesterol levels? Stimulation of glutamatergic transmission was found to induce a loss of cholesterol from synaptic membranes and recruitment of CYP46A1 – an enzyme responsible for cholesterol removal – to the synaptic plasma membrane (Sodero et al., 2012; Brachet et al., 2015; Mitroi et al., 2019). Taken together, these findings highlight the dynamic interplay between cholesterol levels and glutamatergic transmission.

Long-chain PUFAs, particularly DHA, are also found to be enriched in synapses and could play an important role in compartmentalizing the membrane and thereby influencing synaptic transmission. The addition of exogenous DHA to dissociated neuronal cultures was found to enhance spontaneous glutamatergic synaptic activity and promote NMDAR function (Nishikawa et al., 1994; Cao et al., 2009). Furthermore, the protein levels of both AMPAR and NMDAR subunits were higher in the DHA-supplemented cultures (Cao et al., 2009). However, DHA-treatment has been linked to variable effects on synaptic plasticity on brain slices. Exogenous DHA supplementation leads to facilitated LTP in the corticostriatal pathway (Mazzocchi-Jones, 2015) whereas LTP and LTD in the CA1 region were found to be impaired (Young et al., 1998; Mirnikjoo et al., 2001) or unaffected (Fujita et al., 2001; Mazzocchi-Jones, 2015). Some of the discrepancies might arise from region-dependent effects of DHA on synaptic plasticity. In the CA1 region LTP was inhibited whereas in the dentate gyrus there was no effect on LTP after intracerebroventricular injection of DHA (Itokazu et al., 2000). Interestingly, dietary supplementation or deprivation has proven to be an effective method of manipulating DHA levels. The importance of DHA for synaptic plasticity has been found both in young mice, where maternal dietary deprivation of DHA leads to inhibited induction of LTP (Cao et al., 2009), as well as in old rats, where the age-related impairment of LTP is restored by a DHA-supplemented diet (McGahon et al., 1999). Although the variety of results found could be a result of the different experimental paradigms used, it is apparent that DHA plays an important role in modulating cognitive functions. This is highlighted also from the finding that DHA deficiency results in affected spatial learning whereas the *fat-1* transgenic mouse, producing high DHA levels, shows improved spatial learning (Fedorova et al., 2007; He et al., 2009). Lastly, it is important to note that apart from the structural role these lipids can play in membranes, cholesterol (through its metabolites) and PUFAs also have roles as signaling intermediates (Bazinet and Layé, 2014; Petrov et al., 2016). Therefore, although it cannot be concluded from these studies that modulating either cholesterol or PUFA levels solely influences membrane organization, these findings underscore the importance of synaptic membrane composition for neuronal function.

## Additional Layers Contributing to Membrane Organization: Extrinsic Factors

The intrinsic properties of lipids are likely to contribute to membrane organization, but in cellular membranes extrinsic factors add an additional layer of complexity. For instance, interactions with the underlying actin cytoskeleton, oligomerization of membrane proteins or immobilized, membrane-associated protein scaffolds can greatly impact domain formation in the membrane (Kusumi et al., 1993; Fujiwara et al., 2002; Tulodziecka et al., 2016). Particularly at the PSD, that contains a high density of transmembrane and membrane-associated proteins, reciprocal interactions between lipid species and proteins are likely to influence postsynaptic membrane organization.

### Post-translational Lipid Modifications

In addition to hydrophobic structures in proteins, covalent binding of lipidic moieties can mediate the membrane association of proteins (Figure 2). These lipid modifications can be irreversibly added during translation or can be reversibly attached post-translationally by several enzymes [reviewed in detail in Magee and Seabra (2005), Hentschel et al. (2016), Resh (2016)]. Examples of irreversible lipid modifications include myristoylation and prenylation where myristoyl and prenyl groups are attached, respectively. On the other hand, the binding of a GPI anchor or palmitate group are reversible modifications that allow dynamic regulation of protein localization. Many proteins located in the PSD (either transmembrane or membrane-bound) present reversible lipid modifications that can be regulated by activity, incorporating another layer of control of synaptic function. The role of protein palmitoylation in synaptic plasticity is covered more extensively in the following reviews: Fukata et al. (2016), Ji and Skup (2021).

The saturated nature of the lipophilic palmitate group is thought to contribute to the association of palmitoylated proteins with ordered membrane domains. In fact, it has been shown that palmitoylation is essential for partitioning of transmembrane proteins to the ordered domain of GPMVs (Levental et al., 2010; Lorent et al., 2017). Several synaptic receptors are palmitoylated. For instance, different AMPAR subunits are palmitoylated at specific sites (Hayashi et al., 2005). Beyond establishing a quality check-point for protein surface expression, this lipid modification is shown to be a regulated activity-dependent process that controls AMPAR trafficking and recycling (Greaves and Chamberlain, 2007; Yang et al., 2009). Also, NMDAR subunits undergo palmitoylation, influencing their trafficking and stabilization at the synaptic plasma membrane (Hayashi et al., 2009). Nevertheless, it is worth noting that while palmitoylation generally promotes the partitioning of transmembrane proteins into ordered membrane regions, it is not strictly necessary nor sufficient in all cases. For example, the transferrin receptor, a canonical non-raft marker, is palmitoylated at two residues, and the raft reporter caveolin is present in detergent resistant fractions even when its palmitoylation residues are mutated (Alvarez et al., 1990; Dietzen et al., 1995).

For cytosolic proteins, palmitoylation mediates the efficient and dynamic translocation to the membrane. A prominent example is the protein AKAP79, which undergoes dynamic, activity-regulated palmitoylation (Keith et al., 2012; Woolfrey et al., 2015). Interestingly, palmitoylation of AKAP79 is required for its recruitment to dendritic spines and contributes to its stabilization in membranes through association with lipid rafts, which occurs only when it is palmitoylated (Delint-Ramirez et al., 2011; Keith et al., 2012; Purkey et al., 2018). Additionally, the main organizer of PSD architecture, PSD-95, is also anchored to the membrane through palmitoylation of two residues (Topinka and Bredt, 1998; Craven et al., 1999; El-Husseini et al., 2000). Interestingly, Tulodziecka et al. (2016), using biochemical approaches and lipidome analysis of synaptosomal membrane fractions, revealed a developmentally regulated increase in PSD-95 palmitoylation, which is accompanied by an enrichment of domain-promoting lipid species. Thus, while it is clear that palmitoylation controls membrane targeting of key synaptic components, it is plausible that palmitoylation also serves as a nucleation platform for defined lipids. As such, insertion of palmitoylated proteins such as PSD-95, could facilitate the segregation of protein/lipid nanodomains that contribute to the subsynaptic organization of the PSD. In line with this notion, the use of a specific intrabody recognizing palmitoylated PSD-95 in combination with STED microscopy revealed subsynaptic nanodomains of palmitoylated PSD-95 (Fukata et al., 2013). Additionally, PSD-95 palmitoylation regulates its conformation and orientation at the PSD, subsynaptic organization, as well as AMPAR clustering and surface expression at synapses (El-Husseini et al., 2002; Tsutsumi et al., 2008; Fukata et al., 2013; Jeyifous et al., 2016; Yokoi et al., 2016), ultimately controlling synaptic strength.

Although palmitoylation regulates trafficking and membrane targeting of synaptic proteins, its dynamic nature could thus also contribute to regulate the nanoscale distribution of synaptic proteins. Although further experiments are required to elucidate this point, it is tempting to speculate that palmitoyl residues contribute to this subsynaptic organization through interactions with defined membrane regions.

### Protein-Lipid Interactions

Several models of membrane organization include the role of cortical actin in membrane organization (Kusumi et al., 1993; Fujiwara et al., 2002). In particular, the picket-fence model poses that certain actin-linked transmembrane proteins act as “pickets” and hinder diffusion of phospholipids to the next compartment (Figure 2). Even though the actin cytoskeleton is absent from the PSD, it is still one of the major constituents of spines and greatly influences spine morphogenesis and architecture, having a crucial role in neuronal function (Sidenstein et al., 2016; Basu and Lamprecht, 2018). Using single-molecule tracking of a lipid-bound protein Renner M. L. et al. (2009) revealed that actin depolymerization increases diffusion rates of the probe indicating that the actin cytoskeleton could hinder the diffusion of membrane proteins in spines.

Interestingly, several receptors have been reported to contain specific recognition domains for cholesterol and sphingolipids that could be involved in concentrating these receptors in specific lipid domains (Hanson et al., 2008; Jafurulla et al., 2017). For example, mGluR1 is recruited to lipid rafts through a cholesterol recognition/interaction amino acid consensus (CRAC) motif. This recruitment is enhanced upon agonist activation of the receptor, and mutations that reduce mGluR1 affinity for lipid rafts as well as alterations in cholesterol content have a direct effect in the regulation of the agonist-dependent activation of downstream pathways (Kumari et al., 2013). Nevertheless, it is worth noting that although these motifs are present in integral membrane proteins, there is inconclusive evidence to support their necessity or sufficiency for cholesterol binding. Although later efforts have focused on defining a structure-based cholesterol-binding pocket consensus (Marlow et al., 2021), cholesterol and sphingolipids can also interact with membrane-associated proteins and receptors that lack such specific binding motifs. Such lipid-protein interactions could then form a so-called “lipid shell,” allowing proteins to segregate into defined domains (Anderson and Jacobson, 2002; Fantini and Barrantes, 2009). Interestingly, recent structural studies of AMPARs in complex with CNIH2, but not with CNIH3, presented the acyl chains of two lipids penetrating the CNIH-binding site. Therefore, by extending the hydrophobic network and preventing a closer CNIH2-AMPAR interaction, membrane lipids could contribute to regulate receptor function (Zhang et al., 2021). In addition to binding to their specific scaffold proteins, these receptor-specific properties and their interaction with defined PSD membrane regions could also contribute to the segregation of AMPA- and NMDARs on distinct nanodomains within the PSD (Goncalves et al., 2020; Li et al., 2021).

## Technical Advances and Challenges to Study Lipid Organization in the Synaptic Plasma Membrane

The precise organization of different lipid species within the postsynaptic membrane remains largely elusive. This lack of understanding predominantly arises from the lack of adequate tools to study the integrity and lateral heterogeneity of biological membranes in their native state (Jacobson et al., 2007). Nevertheless, new tools continue to be developed to bridge this knowledge gap (Muro et al., 2014; Sezgin et al., 2017) and it will be exciting to see the application of these tools to study the synaptic membrane.

Recent advances in lipidomics methods allow studying the composition of different neuronal compartments in greater detail, including the synaptic plasma membrane (Iuliano et al., 2021). However, while detailed lipidomic characterization provides a general picture of membrane composition (Aureli et al., 2015; Fitzner et al., 2020), it does not reveal the heterogeneity and dynamics of the lateral order of lipids in the membrane. Nevertheless, these studies provide important insights and can be currently combined with *in silico* analysis and databases to analyze protein-membrane interactions to provide further insights into the molecular dynamics at specific membranes (Ingólfsson et al., 2017; Mohamed et al., 2019; Hernández-Adame et al., 2021).

Major advances and efforts have been developed in recent years to directly visualize different lipid species and determine their precise localization and organization. For an in-depth review and overview of fluorescent lipid probes, we refer to Klymchenko and Kreder (2014). A major difficulty in visualizing lipids with fluorescence microscopy arises from the fact that fluorophores are often almost the size of the lipid molecule itself. The addition of such fluorophores could therefore influence the behavior of the lipid and alter its specific amphiphilic properties thereby changing its dynamics. One strategy to circumvent this caveat is to label the head group of the lipid with a fluorophore through the addition of a linker to prevent interaction with the surrounding headgroups (Kinoshita et al., 2017; Mobarak et al., 2018). Such newly developed fluorescent lipid analogs combined with super-resolution imaging (e.g., STED-FCS), single-molecule tracking, and expansion microscopy allow the study of membrane organization at high spatial resolution (Lenne et al., 2006; Eggeling et al., 2009; Mizuno et al., 2011; Klymchenko and Kreder, 2014; Komura et al., 2016; Götz et al., 2020; Sun et al., 2021). Alternatively, reporters that bind to specific lipids can also be used. However, such probes can influence the native membrane organization. For example ChTx, that can bind up to five GM1 gangliosides and thereby could induce cluster formation (Day and Kenworthy, 2015). In the case of cholesterol, filipin is widely used for visualization, but requires fixation because the dye permeabilizes membranes (Behnke et al., 1984). An alternative solution relies on the use of a single domain (D4) from a cholesterol-binding toxin, being sufficient for the binding of cholesterol and use as a sensor for cholesterol in live cells without perturbing its native behavior (Maekawa, 2017).

Single-molecule tracking studies have proven to be a powerful approach in studying the dynamic behavior of lipids and transmembrane proteins in synapses (Choquet and Triller, 2013) and revealed for instance the dynamic exchange of receptors in and out of synapses. Single-molecule trajectories also provide spatial information on the local, temporal confinement of transmembrane proteins, defined as regions where molecules are retained longer than expected from a Brownian moving molecule (Saxton, 1993; Simson et al., 1995), that could indicate the presence of membrane domains. A particularly powerful approach to study the dynamic behavior of lipids is high-speed (up to 25-μs intervals) single-molecule tracking of lipids coupled to photostable dyes. This can reveal temporal subdiffusive behavior and confinement of lipids and membrane proteins that are not observed at typical, slower frame rates (20–30-ms intervals; Fujiwara et al., 2002). Such studies revealed for instance that at these time scales most lipid species and transmembrane proteins undergo short-term confinement in nanoscale compartments and longer-term “hop” movements to adjacent compartments, a phenomenon referred to as “hop diffusion” (Fujiwara et al., 2002; Kusumi et al., 2010). These compartments have been related to the picket-fence model where lipids and transmembrane proteins “hop” from compartments fenced by cortical actin segments (Fujiwara et al., 2016).

The use of environment-sensitive dyes (Danylchuk et al., 2020) allows the study of membrane ordering. These lipophilic dyes have a different emission spectrum dependent on their localization in a more ordered or disordered phase of the membrane. Interestingly, a recent study made it even possible to image lipid order at the nanoscale using the photoswitchable solvatochromic probe NR4A in combination with super-resolution microscopy (Danylchuk et al., 2019). However, some of these probes are derived from voltage-sensitive probes, and could thus behave differently in the excitable membranes of neurons (Obaid et al., 2004).

Finally, a direct test of how individual lipids contribute to membrane organization or functioning in living cells is still lacking. Specific modulation of the composition of cellular membranes cannot be achieved with common pharmacological treatments. Therefore, there is still a void in molecular tools to locally and temporally manipulate membrane composition without affecting downstream pathways. Optical manipulation of lipid biosynthesis might be an interesting future direction to manipulate lipid levels with high spatiotemporal precision (Kol et al., 2019).

Altogether, it is increasingly clear that although recent technical developments provide great insight, a combination of tools and approaches is still required to define the precise lipid composition and organization at the nanoscale in the synaptic membrane.

## Conclusion and Future Directions

The lateral distribution of lipids and proteins in the plasma membrane is highly heterogeneous and is organized as a dynamic patchwork with specific components concentrated in domains that vary largely in size and lifetime. The unique intrinsic composition of the synaptic membrane, as well as the specific spatial distribution of lipids, is predicted to contribute to the formation of specialized nanodomains within the postsynaptic membrane. It will thus be important to understand how lipid and protein components of the postsynaptic plasma membrane interact to contribute to the organization and function of synapses.

Different approaches have been used to characterize the lipid composition of the synaptic plasma membrane. Through isolation of enriched synaptic plasma membrane fractions important observations have been made. In particular, it is evident that the synaptic membrane is enriched in cholesterol and PUFAs and that its composition evolves during development. However, the dynamic nature and lateral heterogeneity of the membrane precludes drawing the complete picture. Therefore, development of new tools is required to provide a better understanding of the organization of the synaptic plasma membrane and how it is modulated by neuronal activity. Importantly, information can be gathered not only on a descriptive level, but also through finer tools to manipulate membrane composition in a spatial and temporally regulated manner.

Another interesting notion is the cooperative nature of protein-lipid interactions. Although synapses have raft properties and general concepts for membrane organization seem to be true for the synaptic membrane, the high abundance of proteins within the synapse makes it a unique structure. Thus, generalizing models of membrane organization and imposing these on how synapses are organized is not straightforward. Nevertheless, while protein-protein domain nucleation undoubtedly plays an important instructive role in shaping the nanoscale architecture of synapses, the interaction of proteins with lipids in the surrounding membrane is likely modulating this nanoarchitecture. This is particularly relevant for proteins containing lipid interacting domains as well as post-translational lipid modifications. The challenge is thus to not only consider protein- and lipid-driven lateral organization as mutually exclusive mechanisms, but to investigate the concerted actions of proteins and lipids in generating the functional heterogeneity of the postsynaptic membrane. However, determining the contribution of lipids to synaptic organization remains a considerable challenge. One important consideration is that lipids can have a dual role, both as structural organizers of membrane domains and as signaling molecules. In fact, lipid signaling greatly contributes to neuronal function (Dotti et al., 2014), directing both intracellular transport of vesicles as well as controlling targeting or activation of key enzymes. For example, lysophospholipid-triggered signaling controls excitatory and inhibitory postsynaptic currents through defined presynaptic and postsynaptic mechanisms, respectively (García-Morales et al., 2015). Thus, altered synaptic function as a result of experimental lipid composition manipulations, could arise from effects on membrane organization, but could also be an indirect effect of disrupted signaling. Designing novel experimental tools to specifically delineate these entangled functions of lipids in synaptic signaling is a formidable task. Nevertheless, the rapid progress in the field of membrane biology and the ongoing efforts in developing novel, specific experimental tools to study membrane organization, are likely to stimulate studies on postsynaptic membrane organization. Such studies are critical to move the field toward a more comprehensive model that integrates both nanoscale protein organization as well as the heterogeneity of the synaptic lipidome.

## Author Contributions

MW, YG, and HM wrote the manuscript. All authors contributed to the article and approved the submitted version.

## Conflict of Interest

The authors declare that the research was conducted in the absence of any commercial or financial relationships that could be construed as a potential conflict of interest.

## Publisher’s Note

All claims expressed in this article are solely those of the authors and do not necessarily represent those of their affiliated organizations, or those of the publisher, the editors and the reviewers. Any product that may be evaluated in this article, or claim that may be made by its manufacturer, is not guaranteed or endorsed by the publisher.
